# Analysis of IL-1**α**(−889) and IL-1B(+3953) Gene Polymorphism in Syrian Patients with Aggressive Periodontitis: A Pilot Study

**DOI:** 10.5402/2011/682564

**Published:** 2011-11-17

**Authors:** Kenan Shibani, Reem Shhab, Razan Khattab

**Affiliations:** Department of Periodontology, Faculty of Dentistry, Damascus University, Damascus, Syria

## Abstract

Polymorphisms in IL-1 gene have been suggested to influence transcription of IL-1*α* and IL-1B and thereby the pathophysiology of periodontitis. Using genotyping IL-1 test, a pilot study was conducted on 32 Syrian patients with aggressive periodontitis (AgP) and 35 healthy controls to investigate the association between the IL-1*α*(−889), IL-1B(+3953) gene polymorphisms and AgP among schoolchildren. The results revealed a similar distribution of genotypes between patients and controls, and did not support an association between IL-1 gene polymorphisms and AgP, however, the association was significant in male patients only. To determine and confirm any susceptible or resistant genes for AgP, future studies should use many target genes and well-defined related periodontal outcomes.

## 1. Introduction

Periodontitis is an opportunistic inflammatory disease of the periodontium. It is widely regarded as one of the most common diseases worldwide. Aggressive periodontitis (AgP) comprises a group of rare periodontal diseases characterized by frequently severe clinical manifestations that affect young individuals, progress rapidly, and can involve multiple family members. Although the presence of bacteria is essential for the onset of the disease, the number and type of these microorganisms are not sufficient to explain differences in the inflammatory and immune responses and consequently, in the severity of the periodontal disease [1]. In recent years, studies have demonstrated that periodontitis is associated with elevated levels of inflammatory cytokines [2], which have a substantial impact on numerous biological activities, and they are taking part in triggering inflammatory cascades and systems [3]. Interleukin-1 (IL-1) is considered a major mediator of periodontal inflammation. It affects nearly every cell type, often in concert with other cytokines or small mediator molecules (e.g., phospholipase A2 type II, cyclooxygenase-2(cox2)). However, the varied biological properties of IL-1 result from its effects on the expression of various genes that regulate the production of the other cytokines [4]. IL1 modulates extracellular matrix components, enhances bone resorption in the periodontal tissues, stimulates fibroblasts and other nucleated cells to produce matrix metalloproteinase, activates plasminogen, and triggers prostaglandin synthesis [5]. IL-1 also strongly stimulates connective tissue catabolism, activates immunocytes, and regulates adhesion molecules that facilitate migration of leukocytes into tissues [6]. IL-1 family genes are located in a cluster on human chromosome 2q13, a specific genotype in the IL-1 cluster that includes a specific locus is associated with increased IL-1 production and increased susceptibility to periodontitis [7].

It has currently become evident that in the case of most diseases other factors exist, and those factors do not cause disease but they modify its course, rendering it more severe. Among these factors are genetic alterations, called polymorphisms, which are commonly found in the population [8]. Gene polymorphisms are locations within the genome that vary in sequence between individuals and are very prevalent, affecting at least 1% of the population [9]. Many genes responsible for cytokine production exhibit polymorphisms which can modify the production of cytokines such as IL-1 [10]. Polymorphisms in various cytokine genes can influence the level of secretion of these substance and explain the variations in individual immune-inflammatory responses to a bacterial virulence [8]. Recent studies have indicated that the IL-1 gene polymorphism might be associated with a greater severity of the disease in patients with AgP [11]. More than 99% of the African American patients of localized juvenile periodontitis (LJP) exhibit polymorphism of IL1B(+3953) allele 1 [12]. There was a clear influence of the polymorphism of genes IL-1B(+3954), IL-1*α*(−889), and IL6(−147) on the AgP susceptibility [13]. On the other hand, there was not any significant association of the polymorphism of IL-1genes with the AgP infection in Japan [14].

The objective of the present study was to investigate the association between the IL-1*α*(−889), IL-1B(+3953) gene polymorphisms and aggressive periodontitis among schoolchildren in Syria.

## 2. Materials and Methods

The study protocol was approved by the Research Ethics Committee of the Faculty of Dentistry, Damascus University. Sixty seven students distributed into AgP patients and healthy controls having been selected from similar geographical areas, had a similar socioeconomic status, participated in the study after providing informed consent and being advised about their disease. Evaluation of each participant consisted of personal and family medical and dental history, panoramic radiographs, and periodontal examinations. Subjects were free of systemic diseases, and those who were smokers used anti-inflammatory or antibiotic drugs in the last three months, or used orthodontic devices were excluded. Moreover, all of participants had at least 24 teeth.

The AgP group is comprised of 32 Syrian patients (11 males and 21 females), aged 12–20 years (mean = 14.6), from the Department of Periodontology, Faculty of Dentistry, Damascus University. A clinical diagnosis of LAgP was made for 8 patients, while 24 patients were diagnosed as having GAgP according to the criteria of the IWC 1999 International Workshop for a Classification of Periodontal Diseases and Conditions [15]. The following clinical parameters: probing depth (PD), clinical attachment loss (CAL), and bleeding on probing (BOP) were recorded at six sites (mesiobuccal, buccal, distobuccal, mesiolingual/palatal, palatal/lingual, distopalatal/lingual) for each tooth using a Williams Periodontal Probe (Carl Martin, Solingen, Germany), while Gingival index (GI), and plaque index (PI) were recorded at four sites (mesiobuccal, buccal, distobuccal, lingual/palatal).

The control group consisted of 35 healthy students (20 males and 15 females), aged 12–20 years (mean = 15.3), and who do not have a clinical evidence of attachment loss in any tooth after recording (CAL, PD, BOP) indices at the previous six sites.

## 3. Sample Collection and Genotyping Analysis

One buccal swab sample was obtained from each individual by moving it on the interior surface of the cheek for 20–30 seconds, extra saliva was dried by using a hand fan for one minute. Genotyping IL-1 tests were achieved in cooperation with the Hain Lifescience Laboratory, Germany, and based on the DNA*·*STRIP technology which permits the combined characterization of polymorphisms at position (−889) of the human (IL-1*α*) gene, and position (+3953) of the human (IL-1B) gene. The whole procedure was divided into three steps: DNA extraction from a patient sample (buccal swab), a multiplex amplification with biotinylated primers, and a reverse hybridization.

The hybridization included the following steps: chemical denaturation of the amplification products, hybridization of the single-stranded, biotin-labeled amplicons to membrane-bound probes, stringent washing, addition of a streptavidin/alkaline phosphatase (AP) conjugate, and an AP- mediated staining reaction.

## 4. Statistical Analysis

To determine whether any significant differences in polymorphism frequencies occurred between the AgP and control populations, and according to both genders, data analyses were performed using computer program (SPSS 10.0 for Windows, Chicago, Ill, USA). We compared allele and genotype frequencies, using Fisher's exact test. The strength of the associations was determined using odds ratio (OR) calculations and 95% confidence intervals (CIs).

## 5. Results

AgP patients had a mean probing depth of 5.1 ± 1.1 mm and clinical attachment loss of 6.8 ± 2.6 mm (at affected teeth); control individuals had a mean probing depth of 1.8 ± 0.5 mm and without any clinical attachment loss.

The homozygous IL-1*α*(−889) allele 1 was present in 57.1% of the control group and in 37.5% of AgP group, homozygous IL-1*α*(−889) allele 2 was present in 8.6% of the control group and in 12.5% of AgP group, while the heterozygous IL-1*α*(−889) allele (1,2) was present in 34.3% of the control group and in 50% of AgP group. The differences between the two groups were not significant as analyzed with Fisher's Exact test (*P* = 0.274), Table 1.

The frequency of IL-1*α*(−889) allele 1 and allele 2 was 74.3% and 25.7%, respectively, in control group, and in the AgP group the frequency for allele 1 was 62.5% and 37.5% for allele 2. The differences between two groups were not significant; OR for IL-1*α*(−889) allele 2 in the AgP group was 1.733 (95% CI (0.829–3.623), *P* = 0.19), Table 1.

However, for IL-1B(+3953), genotype frequencies in the control group were 62.8% for genotype (1,1), 22.9% for genotype (2,1) (heterozygous), and 14.3% for genotype (2,2). Statistically similar genotype frequencies were obtained for the AgP groups: 46.9% for genotype (1,1), 43.7% for genotype (2,1), and 9.4% for genotype (2,2). The differences between two groups were not significant as analyzed with Fisher's Exact test (*P* = 0.194), Table 2.

The frequency of allele 1 and allele 2 was 74.3% and 25.7% in control group, and in the AgP group the frequency for allele 1 was 68.8%, and for allele 2 was 31.2%. The differences between two groups were not significant; OR for IL-1B(+3953) allele 2 in the AgP group was 1.313 (95% CI (0.619–2.788), *P* = 0.566), Table 2.

The homozygous IL-1*α*(−889) allele 1 was present in 60% of the males in control group and in 9.1% of the males in AgP group, homozygous IL-1*α*(−889) allele 2 was present in 5% of the males in control group and in 18.2% of the males in AgP group, while the heterozygous IL-1*α*(−889) allele (1,2) was present in 35% of the males in control group and in 72.7% of the males in AgP group. The differences between two groups were significant as analyzed with Fisher's Exact test (*P* = 0.012), Table 3.

The frequencies of IL-1*α*(−889) genotypes among the females in AgP and control groups were very similar, and the comparison revealed no significant association as analyzed with Fisher's Exact test (*P* = 1.0), Table 3.

The homozygous IL-1B(+3953) allele 1 was present in 75% of the males in control group and in 27.3% of the males in AgP group, homozygous IL-1B(+3953) allele 2 was present in 15% of the males in control group and 9.1% of the males in AgP group, while the heterozygous IL-1B(+3953) allele (1,2) was present in 10% of the males in control group and in 63.6% of the males in AgP group. The differences between two groups were significant as analyzed with Fisher's Exact test (*P* = 0.006), Table 4.

The frequencies of IL-1B(+3953) genotypes among the females in AgP and control groups were very similar, and the comparison revealed no significant association as analyzed with Fisher's Exact test (*P* = 0.895), Table 4.

## 6. Discussion

Aggressive periodontitis is a multifactorial disease resulting from the complex interactions between the host, microorganisms, and environment. In recent years, studies have demonstrated that periodontitis is associated with elevated levels of a variety of inflammatory biomarkers. Furthermore, genetic variants of some cytokines confer susceptibility to periodontitis.

The prevalence of AgP among young Syrian population was reported to be 2.8%, and considered very high when compared to the other populations [16]. Therefore, 32 Syrian AgP patients and 35 healthy control individuals were recruited. The AgP and control groups were similar regarding age distribution. Nonsmokers and former smokers were included since smoking has been identified as the major environmental risk factor associated with increased incidence and severity of periodontitis [17].

Genetic studies of world populations support the categorization into five major groups: Africans, Caucasians, Pacific Islanders, East Asians, and Native Americans [18]. Furthermore, population clusters identified by genotype analysis seem to be more informative than those identified by skin color or self-declaration of race [19].

 Syrians belong to the Caucasian group, and the literature contains no other data regarding the association of IL-1gene polymorphisms with AgP in Syrian population. The allele and genotype distributions observed in the both groups were similar to those previously reported in Caucasian populations. However, our results demonstrated that there was no significant association between AgP and the IL-1*α*(−889) and IL-1B(+3953) gene polymorphisms in patients with AgP. 

The findings presented here disagree with the results provided by Brett et al. [20] which pointed to a strong evidence of association between IL-1B(+3953) gene polymorphism and AgP. Guzeldemir et al. [21] reported that IL-1 gene polymorphisms appeared to have a role in susceptibility to LAgP in the Turkish population. The present results agree with those reported by other investigators studying patients with AgP. For example, Gonzales et al. [22] failed to detect an association with IL-1 genotypes in European Caucasians and Central Americans with AgP. Also Scapoli et al. [23] did not support the existence of a causative variant for generalized AgP within the 2q13-14 region in an Italian Caucasian population. Finally, Fiebig et al. [24] did not report an association between variants in the IL-1 gene cluster and AgP in northern European Caucasian patients.

The results showed a significant association between AgP and the IL-1*α*(−889) and IL-1B(+3953) gene polymorphisms in male patients only, which indicates that males were more sensitive to the genetic variations, and this could be attributed to the differences in prevalence of genetic polymorphisms between both genders. This result corresponds with the study of Gera and vári [25] which found that the association between different candidate gene polymorphism and its periodontal effects is still very controversial, and certain associations are dependent on sex and race.

In literature, AgP was generally found to be more common in females than males, and this might be explained by the fact that females tend to be more concerned to their appearance and more likely to seek dental attention than males. Another possible reason is the earlier age of puberty in females; hormonal changes during the menstrual cycle and pregnancy might aggravate the clinical course of the disease [26]. While many studies have shown the association between the influence of gene polymorphisms on many other diseases and the gender, for example, Queiroz et al. [27] reported that increasing age, male sex, and IL1 gene polymorphisms were independently associated with duodenal ulcer. However, no confident data are available about the above- mentioned association in periodontal diseases, which necessitates a study of the relation between sex and the impact of gene polymorphisms on AgP.

The difficulty in associating gene polymorphisms with AgP might be explained by the lack of higher expression of a single gene in the disease. Probably, there are other genes altering the expression of genes and influencing the clinical expression of the disease. Furthermore, multiple polymorphisms might be necessary for an increase in the severity of the disease. In addition, specific genes may vary among different populations and/or ethnic groups, and true heterogeneity in the susceptibility to the disease might be present.

In conclusion, the present study revealed no association between the IL-1*α*(−889) and IL-1B(+3953) gene polymorphisms and AgP in Syrian population, in the absence of the other risk factors, whilet the results indicate the need to future studies which may contribute to the investigation of the effect of sex on the previous associations.

## Figures and Tables

**Table 1:** Frequency of IL-1*α*(−889) genotype and allele in AgP patients and healthy controls.

IL-1*α*(−889)	Group			
	AgP		Control	
	*N*	%	*N*	%
1,1	12	37.5	20	57.1
2,2	4	12.5	3	8.6
1,2	16	50	12	34.3

Total	32	100	35	100
Allele 1	40	62.5	52	74.3
Allele 2	24	37.5	18	25.7

Total	64	100	70	100

**Table 2:** Frequency of IL-1B(+3953) genotype and allele in AgP patients and healthy controls.

IL-1B(+3953)	Group			
	AgP		Control	
	*N*	%	*N*	%
1,1	15	46.9	22	62.8
2,2	3	9.4	5	14.3
1,2	14	43.7	8	22.9

Total	32	100	35	100
Allele 1	44	68.8	52	74.3
Allele 2	20	31.2	18	25.7

Total	64	100	70	100

**Table 3:** Distribution of IL-1*α*(−889) genotype in AgP patients and healthy controls according to the gender.

IL-1*α*(−889)	Male				Female			
	AgP		Control		AgP		Control	
	*n*	%	*N*	%	*N*	%	*n*	%
1,1	1	*9.1	12	60	11	52.4	8	53.3
2,2	2	*18.2	1	5	2	9.5	2	13.3
1,2	8	*72.7	7	35	8	38.1	5	33.3

Total	11	100	20	100	21	100	15	100

**Table 4:** Distribution of IL-1B(+3953) genotype in AgP patients and healthy controls according to the gender.

IL-1B(+3953)	Male				Female			
	AgP		Control		AgP		Control	
	*n*	%	*n*	%	*n*	%	*n*	%
1,1	3	**27.3	15	75	12	57.1	7	46.7
2,2	1	**9.1	3	15	2	9.5	2	13.3
1,2	7	**63.6	2	35	7	33.3	6	40

Total	11	100	20	100	21	100	15	100

**P < 0.01.
